# Colorimetric and Fluorimetric DNA Detection with a Hydroxystyryl–Quinolizinium Photoacid and Its Application for Cell Imaging

**DOI:** 10.1002/chem.201903017

**Published:** 2019-09-09

**Authors:** Avijit Kumar Das, Sergey I. Druzhinin, Heiko Ihmels, Mareike Müller, Holger Schönherr

**Affiliations:** ^1^ Department of Chemistry and Biology, and Center of Micro- and Nanochemistry and Engineering (*Cμ*) University of Siegen Adolf-Reichwein-Str. 2 57068 Siegen Germany

**Keywords:** bioorganic chemistry, fluorescent probes, heterocycles, live-cell imaging, photoacids

## Abstract

The combination of styryl dye properties with the acidity and strong photoacidity of the 2,2′‐[(1′′‐hydroxy‐4′′‐methyl‐(*E*)‐2′′,6′′‐phenylene)]‐bisquinolizinium enables the detection of DNA by distinct absorption and emission color changes and the fluorimetric detection of DNA in cells with epifluorescence and confocal fluorescence microscopy.

## Graphical abstract

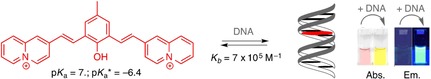

Nucleic acids are essential biomacromolecules that constitute key targets in biochemistry, pharmaceutical sciences and medicine, so that their rapid and straightforward detection is still an important task.^1^ In particular, the application of optical methods for DNA staining, specifically with fluorescent organic dyes,^2^ figures as a versatile and convenient approach towards the qualitative and quantitative detection of DNA in homogeneous solution and in cells.^3^ To this end, fluorescent light‐up probes whose very low emission in aqueous solution increases upon association with DNA turn out to be useful analytical tools.^4^ Among the classes of organic dyes that may be used for this purpose, styryl dyes have been shown to be an easily available and modifiable class of compounds.^5^ Specifically, the emission intensity of these dyes increases when bound to DNA because the radiationless deactivation by structural changes of the excited styryl unit is efficiently suppressed in the respective binding sites.^6^ In this context, it has been demonstrated that the combination of the established DNA‐binding quinolizinium ion^7^ with styryl dye units results in DNA ligands with remarkable light‐up effects.^8^ At the same time, the weakly acidic and strongly photoacidic hydroxy‐substituted quinolizinium ions exhibit pH‐dependent DNA‐binding properties with significantly changing optical properties in host‐guest complexes.^9^ Based on these results, we proposed that hydroxy‐functionalized styrylquinolizinium derivatives should change their emission properties upon association with DNA, so that they may be used for fluorimetric DNA detection. Herein, we demonstrate with the hydroxy‐phenylene‐bisquinolizinium **3 a**, and by comparison with its *O*‐methylated derivative **3 b**, that the combination of styryl dye properties with the acidity of the derivative enables the staining of DNA by distinct changes of absorption and emission color.

The hydroxy‐ and methoxy‐substituted styrylquinolizinium derivatives **3 a** and **3 b** were synthesized by a base‐induced condensation of 2‐methylquinolizinium (**1**) with the 5‐methylisophthalaldehyde derivatives **2 a** and **2 b** in 70 % and 60 % yield (Scheme 1). The compounds were identified and characterized by NMR spectroscopy, mass spectrometry and elemental analysis. The quinolizinium derivatives **3 a** and **3 b** display a dominant absorption band with a maximum at 376 nm (**3 a**) and 378 nm (**3 b**) in water. Compound **3 a** also exhibits an additional broad, red‐shifted band at 520 nm. Whereas **3 a** only has a very weak emission (*Φ_fl_* < 0.01) at 460 nm in H_2_O, a more intense band was observed for the derivative **3 b** at 530 nm with moderate emission quantum yield (*Φ_fl_*=0.10).

**Scheme 1:**
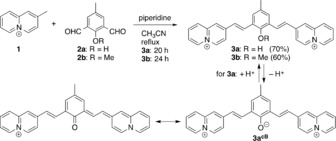
Synthesis of phenylene‐bis(styrylquinolizinium) conjugates **3 a** and **3 b** and prototropic equilibrium of **3 a**.

The pH dependence of the absorption and emission properties of derivative **3 a** were studied with spectrometric acid‐base titrations (Figure 1). From pH 2.4 to 5.2, **3 a** exhibits a long‐wavelength absorption band at 385 nm. With increasing pH of the solution (pH >5), however, the intensity of the latter absorption band decreased, and a red‐shifted broad band developed with a maximum at 520 nm. During the titration, an isosbestic point was formed at 442 nm. This development of bands is characteristic of hydroxyquinolizinium derivatives^10^ and indicates the prototropic equilibrium between **3 a** and its conjugate base **3 a^cB^**. Thus, the short wavelength absorption maximum in acidic medium corresponds to **3 a**, whereas the red‐shifted absorption maximum formed at higher pH values is assigned to **3 a^cB^**. The red shift results from the more pronounced donor‐acceptor interplay between the oxyanion substituent and the quinolizinium unit (Scheme 1).^10^ Further analysis of the titration curve revealed a p*K*_a_ value of 7.06±0.01 (Figure 1 A). Notably, the emission spectrum of **3 a** showed essentially the same weak, very broad emission band at 450 nm between pH 2 and 10 (cf. Figure S3 in the Supporting Information). In contrast, a significant fluorescence enhancement was observed at very strong acidic conditions in aq. HClO_4_ solution. With increasing concentration of HClO_4_, an emission band appeared at 520 nm which was further blue shifted to 505 nm (at 11.8 m) with a strong increase of emission intensity (Figure 1 B). The absorption and emission spectra of **3 a** in acidic and alkaline media were used to estimate the p*K*_a_^*^ value as a measure of excited‐state acidity with Förster‐cycle calculations,^11^ that gave a p*K*_a_* value of −6.4 (cf. Figure S2). This remarkably high excited‐state acidity may be explained by the formation of the stabilized merocyanine‐type structure of **3 a^cB^** in the excited state (Scheme 1), as shown also for resembling superphotoacids.^12^ In contrast to **3 a**, the absorption and emission properties of the methoxy‐substituted derivative **3 b** are essentially independent of the pH of the solution (cf. Figure S4).

**Figure 1:**
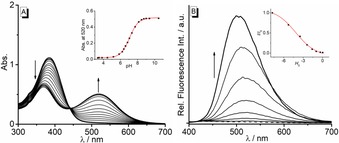
A: Absorption spectra of **3 a** (*c=*20 μm) in Britton‐Robinson buffer; arrows in A indicate the development of absorption from pH 2.0 to 10.5. Inset: Plot of absorption at 520 nm vs. pH; the line indicates the best fit of the experimental data to the theoretical isotherm. B: Fluorescence spectra of **3 a** (*λ*_ex_=376 nm), in aq. HClO_4_ at varied H^+^‐concentration. Inset: Plot of rel. fluorescence intensity vs. *c*(HClO_4_) given as Hammett acidity values *H*_0_ (Ref. 13).

To examine whether the low emission quantum yields of derivatives **3 a** and **3 b** are caused by conformational movement in the excited state, the fluorescence spectra were recorded in media of high viscosity, namely in water‐glycerol mixtures. In viscous solution the conformational changes of an excited molecule are significantly suppressed and thus slower than emission, so that the fluorescence quantum yield increases.^14^ Indeed, with increasing viscosity, as adjusted with rising content of glycerol (wt.% glycerol: 0, 50, 100 %), the low emission intensity of **3 a** and **3 b** in water increased significantly up to *Φ_fl_*=0.33 (**3 a**) and *Φ*_fl_=0.46 (**3 b**) in pure glycerol (cf. Figure S1), which indicates that conformational changes in the excited state contribute mainly to the radiationless deactivation, presumably along with a minor quenching pathway by interactions of the excited molecule with water.

The interactions of the ligands **3 a** and **3 b** with calf thymus (ct) DNA were studied by photometric and fluorimetric titrations in BPE buffer solution (Figure 2). Upon titration of ct DNA to **3 a**, the absorption band of **3 a** at 376 nm decreased and two new bands developed at 383 nm and 420 nm. The absorption maximum at 520 nm decreased also steadily during the DNA titration and almost disappeared. Remarkably, the color change during the titration can be clearly followed by the naked eye (Figure 2 A). It should be emphasized that such a pronounced change of *absorption* color on DNA binding is rarely observed. In the case of **3 b**, the titration of ct DNA led to a hyperchromic effect along with a red shift of the absorption maximum to 388 nm (Δ*λ*=8 nm) (cf. Figure S6). The resulting binding isotherms from the photometric titrations were used to determine binding constants *K_b_* of 7±1×10^5^ m ^−1^ (**3 a**) and 1.86±0.02×10^4^ m ^−1^ (**3 b**). The emission intensity of **3 a** and **3 b** increased significantly in the presence of ct DNA (cf. Figure 2 B). Thus, the addition of ct DNA to **3 a** and **3 b** resulted in an increase of the emission intensities at 515 nm by a factor of ca. 200 (**3 a**, *Φ_fl_*=0.39) and 7 (**3 b**: *Φ_fl_*=0.55) (cf. Figure S8). In the case of **3 a**, an additional very weak emission signal appeared at 720 nm on addition of ct DNA, Overall these titrations showed the characteristic signatures of a DNA binding ligand.^15^ Although at the start of the titration a significant amount of conjugate base **3 a^cB^** is present in solution, it is almost exclusively the ligand **3 a** that binds to DNA, as indicated by the formation of absorption and emission bands, which are assigned to this species, and by the almost complete disappearance of absorption and emission bands of **3 a^cB^**. Most likely, **3 b** has a much higher affinity toward DNA because in this form two cationic charges are still maintained that contribute significantly to the overall binding energy.^16^ In this context, the weak emission signal at 720 nm formed during fluorimetric titration may indicate the aggregation of **3 a^cB^** molecules along the DNA backbone.

**Figure 2:**
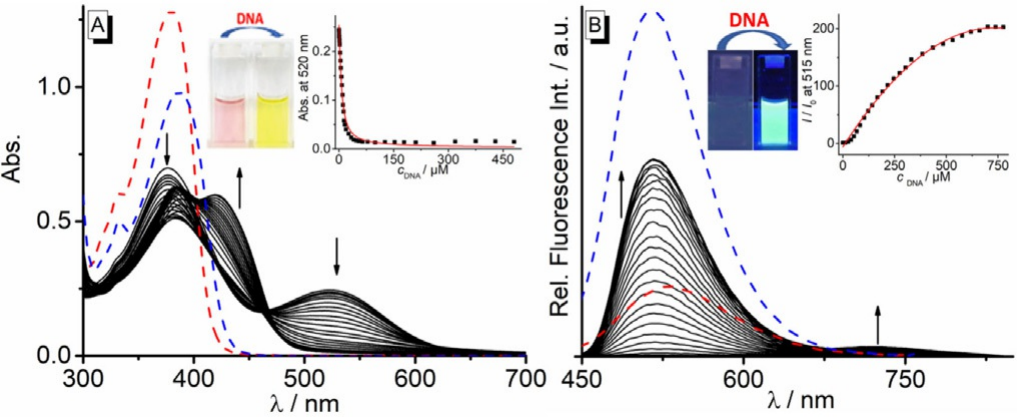
Photometric (A) and fluorimetric titration (B) of **3 a** with ct DNA (*c=*2.7 mm in base pairs) in BPE buffer (10 mm, pH 7.0; with 5 % v/v DMSO, *c*_**3** ***a***_ **=**20 μm); *T=*20 °C; *λ*_ex_=440 nm. Arrows indicate the changes of bands on addition of DNA. Insets A: Picture of absorption (A) and emission (B) color of **3 a** with and without DNA, and plot of the absorption (A) or relative emission (B) vs. *c*_DNA_. Dashed lines: Absorption (A) and emission (B) spectra of **3 b** in absence (red) and presence (blue) of ct DNA.

The association of **3 a** and **3 b** with ct DNA also explains the increasing emission intensity during titration, because within the binding site of the DNA the ligands experience a restricted conformational freedom of movement.^17^ It was demonstrated with the viscosity dependence of the emission intensity of **3 a** and **3 b** (cf. SI) that the low emission quantum yield in water is mainly caused by conformational relaxation in the excited state. Therefore, we propose that the emission light‐up effect of DNA‐bound ligands **3 a** and **3 b** is the result of restricted conformational flexibility that suppresses this radiationless deactivation of the excited state, as has been shown already for several styryl‐ and stilbene‐type dyes.^8^ The fluorescence light‐up effect of **3 a** is about 30 times stronger than the one of **3 b**, presumably because in this case also the deactivation of the excited molecule by prototropic reactions—that leads to much lower emission quantum yields in water—is suppressed within the binding site. Overall, it is demonstrated with these experiments that the derivative **3 a** may be employed as optical probe that indicates DNA by a color change of the absorption *and* a strong emission light‐up effect.

The interactions between the derivatives **3 a** and **3 b** and ct DNA were also examined with CD‐ and LD‐spectroscopy (Figure 3, cf. SI).^18^ The binding of **3 b** with ct DNA resulted in the development of a positive CD band and a negative LD band in the absorption range of the ligand. This development of bands is consistent with an intercalative binding mode.^18^ Specifically, the negative LD band in the absorption range of the ligand unambiguously indicates the intercalated ligand. In contrast, the CD‐ and LD‐spectroscopic data revealed a dependence of the binding mode of **3 a** on the ligand‐DNA ratio (*LDR*). Thus, at higher *LDR* (0.8–1.0), a broad negative LD band appeared with a maximum at 380 nm, that is—alike **3 b**—assigned to the intercalated ligand. At lower *LDR* (0‐0.6), however, a positive LD band at 410 nm was observed, which usually indicates groove binding.^18^ But as the CD signature of the **3 a**‐DNA complex does not change significantly during titration and even maintains its general band structure, we propose that only the orientation of the ligand in the binding site changes, and not the overall binding mode. It is assumed that **3 a** is generally an intercalator, as shown by comparison with **3 b**. At lower *LDR* values, that is, when the DNA binding sites are only partially occupied with ligands, one quinolizinium unit of **3 a** is intercalated whereas the remaining part of the molecule adopts a conformation that enables its accommodation in the neighboring groove. In this binding mode, the intercalated styrylquinolizinium unit gives a negative LD signal whereas the one in the groove leads to a stronger positive signal, which eventually results in the observed positive band. This binding situation is supported by the photometric titration, because with decreasing *LDR* two separate long‐wavelength bands develop instead of the one expected for **3 a**, indicating the two different decoupled styrylquinolizinium units. With increasing *LDR*, however, less space is available for binding along the DNA groove, so that fewer intercalated molecules have the opportunity for the additional partial association in the groove, so that eventually the negative LD band of the intercalated quinolizinium dominates. Obviously, the ligand **3 b** does not have the propensity for this additional groove binding, presumably because of steric hindrance by the methoxy group, as opposed to the hydroxy functionality in **3 a** that may even be employed for hydrogen bonding in the groove.

**Figure 3:**
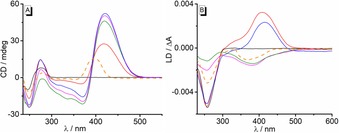
CD (A) and *flow*‐LD spectra (B) of ct DNA (*c=*50 μm) in the absence and presence of **3 a** at *LDR*=0 (black), 0.3 (red), 0.5 (blue), 0.8 (magenta), 1.0 (green) in BPE buffer solution (10 mm, pH 7.0; with 5 % v/v DMSO). Dashed lines: CD and LD spectra of **3 b** in presence of DNA at LDR=1.

The potential of derivative **3 a** to stain cellular DNA was examined by epi‐ and confocal fluorescence microscopy in living (S18) and fixed NIH 3T3 mouse fibroblasts (Figure 4 and Figure 5). In epifluorescence images a clear fluorimetric staining of the nucleus was observed in both cases in both the green and the red channel, as confirmed by co‐localization with Hoechst 33 258 (Figure 4 A and 4 D; cf. Figure S16). Notably, these images showed a characteristic granulated pattern in the nucleus with a strong fluorescent staining of the condensed and non‐active heterochromatin.^19^ The staining of **3 a** was not as bright as that of Hoechst 33258, but it still resulted in a reproducible clear and stable green fluorescence signal of the nucleus with insignificant cell specific autofluorescence background (cf. Figure S17). Remarkably, **3 a** also exhibited a weaker red emission signal with a diffuse signal pattern concentrated at the perinuclear region of the cytoplasm and in the nucleus. However, the brightness of the red emission in the cytoplasm in some cells was even stronger than the negligible green emission and exhibited gaps in the nuclear region of single cells (Figure 4; cf. Figure S18).

**Figure 4:**
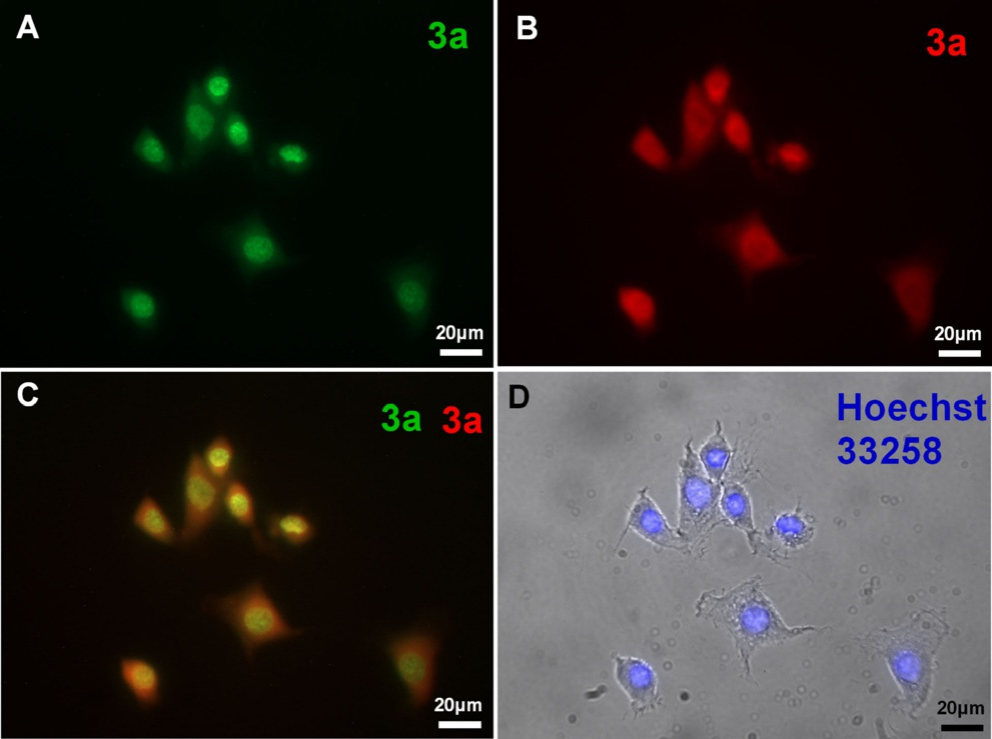
Exemplary epifluorescence microscopy images of NIH 3T3 mouse fibroblasts after fixation and staining with **3 a** (2.5 μm in PBS) for 1 h and counterstained with Hoechst 33258. Pictures show pseudo coloring of the fluorescent emission resulting from three different filter sets. A: *λ*_ex_=450–490 nm, *λ*_em_>515 nm (green channel). B: *λ*_ex_=540–552 nm, *λ*_em_>590 nm (red channel). C: Overlay of red and green channel. D: Overlay of bright field microscopy pictures and nuclear DNA stain with Hoechst 33258 in the blue channel: *λ*_ex_=320–390 nm, *λ*_em_=420–470 nm. Labeling **3 a** stands for both the ligand **3 a** and its conjugate base **3a^cB^** compound. Scale bar: 20 μm.

The cells stained with derivative **3 a** were examined in detail by time‐resolved confocal fluorescence microscopy with *λ*_ex_=485 nm, leading to an excitation of both **3 a** and **3 a^cB^**, with somewhat preferential excitation of **3 a^cB^** (Figure 2, left). The imaging at the fluorescence bands of **3 a** (Figure 5, left) and **3 a^cB^** (Figure 5, right) shows that the cell nucleus and the cytoplasm including the filopodia are stained by these forms, however, the staining is more uniform in the case of **3 a^cB^**. Direct excitation of only **3 a^cB^** at 540–552 nm (see, Figure 1 A) gives a diffuse cell image in Figure 4 B similar to that in Figure 5, proving that **3 a^cB^** is the staining form of **3 a** in the red channel. The fluorescence decays of **3 a** and **3 a^cB^** within the cells are triple‐exponential with decay times *τ*=3.6 ns (26 %), 1.5 ns (53 %) and 0.39 ns (21 %) for **3 a** and *τ*=2.6 ns (39 %), 0.89 ns (40 %) and 0.30 ns (21 %) for **3 a^cB^** and practically no contribution from cell autofluorescence (*cf*. Figure S19). Not matched τ values and their positive amplitudes (*cf*. Figure S19) for the green and red channels indicate that the emission of **3 a^cB^** mainly originates from the direct excitation of **3 a^cB^**, and not from **3 a** deprotonated in the excited state.^20^

**Figure 5:**
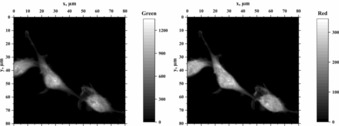
Confocal fluorescence microscopy (cf. Ref. 8b) images of NIH 3T3 mouse fibroblasts after fixation and incubation with **3 a** (2.5 μm) for 1 h; *λ*_ex_=485 nm, *λ*_em_=515–652 nm (left) and 652–732 nm (right).

Interestingly, the subtraction of the red‐channel image (scaled by a factor of 2) from the green‐channel data substantially reduces the cytoplasm fluorescence in the resulting difference image (cf. Figure S20). Based on this observation, together with the result that the component with *τ*=1.5 ns possesses the highest contribution to the overall fluorescence of the stained cell, we conclude that this particular component is associated with the emission of DNA‐bound **3 a**. These staining assignments of **3 a** and **3 a^cB^** are supported by the fluorescence spectra of **3 a** in solution (Figure 2). Although the distinct photophysical properties have to be determined in more detail, these preliminary results from confocal fluorescence microscopy demonstrate that a lifetime‐based fluorimetric analysis of cells can be performed with dyes such as **3 a**.

In summary, we discovered a novel styrylquinolizinium dye whose combination of acidic, strongly photoacidic and DNA‐binding properties may be used for photometric and fluorimetric DNA detection. Strikingly, the prototropic equilibrium between the ligands **3 a** and **3 a^cB^** along with their different absorption colors and different DNA‐binding properties enables the colorimetric detection of DNA. And we have also shown that the emission light‐up effect of the DNA‐bound dye may be used for fluorimetric cell analysis. It is therefore concluded that this type of dye is a promising starting point for the development of efficient and versatile colorimetric and fluorescent DNA stains. For example, additional recognition elements may be introduced to accomplish sequence‐specific DNA binding and detection as observed for similar thiazole‐based bis‐styryl‐type ligands.6d

## Conflict of interest

The authors declare no conflict of interest.

## Supporting information

As a service to our authors and readers, this journal provides supporting information supplied by the authors. Such materials are peer reviewed and may be re‐organized for online delivery, but are not copy‐edited or typeset. Technical support issues arising from supporting information (other than missing files) should be addressed to the authors.

Supplementary
