# Supplementary material for: Colorimetric and Fluorimetric DNA Detection with a Hydroxystyryl–Quinolizinium Photoacid and Its Application for Cell Imaging
Source: Chemistry. 2019 Sep 9;25(55):12703–7. doi: 10.1002/chem.201903017 (PMC6790585; doi:10.1002/chem.201903017)
Supplement: Supplementary file 1 — Supplementary [file CHEM-25-12703-s001.pdf]

CHEMISTRY
A European Journal

Supporting Information

Colorimetric and Fluorimetric DNA Detection with a Hydroxystyryl–Quinolizinium Photoacid and Its Application for Cell Imaging

Avijit Kumar Das, Sergey I. Druzhinin, Heiko Ihmels,* Mareike Müller, and Holger Schönherr[a]

chem_201903017_sm_miscellaneous_information.pdf

Supporting Information

Table of Contents

1.	Equipment	S2
2.	Materials	S2
3.	Synthesis	S2
4.	Absorption and emission spectra in media with different viscosity	S4
5.	Acid-base titrations	S4
6.	Determination of fluorescence quantum yield	S7
7.	Photometric and fluorimetric DNA titrations	S7
8.	CD- and LD-spectroscopic experiments	S10
9.	NMR spectra	S11
10.	NIH 3T3 cell culture and fluorescence staining	S14

1. Equipment

Absorption spectra were recorded with a Varian Cary 100 Bio spectrophotometer in quartz cells (10 mm x 10 mm) with baseline correction, and emission spectra were collected with a Varian Cary Eclipse spectrophotometer in quartz cells (10 mm x 10 mm) at 20 °C. NMR spectra were recorded with a Varian VNMR-S 600 (^{1}H: 600 MHz, ^{13}C: 150 MHz) at 25°C.(DMSO-d_6). Spectra were processed with the software MestReNova and referenced to the residual solvent signals of DMSO-d_6 (^{1}H: δ = 2.50, ^{13}C: δ = 39.5). Elemental analyses data were determined with a HEKAtech EUROEA combustion analyser. ESI mass spectra were recorded on a Finnigan LCQ Deca (U = 6 kV; working gas: Argon; auxiliary gas: Nitrogen; temperature of the capillary: 200 °C). Circular-dichroism (CD) and *flow*-linear-dichroism (LD) spectra were recorded with a Chirascan CD-Spectrometer, Applied Photophysics. The melting points were measured with a BÜCHI 545 (BÜCHI, Flawil, CH).

2. Materials

2-Methylquinolizinium (**1**), 2-hydroxy-5-methylisophthalaldehyde (**2a**), and 2-methoxy-5-methylisophthalaldehyde (**2b**) were synthesized according to the published procedure.[1] Calf thymus DNA (ct DNA, type I; highly polymerized sodium salt; Ɛ = 12824 cm^{-1} M^{-1}) was purchased from Sigma Aldrich (St. Louis, USA) and used without further purification. The ct DNA was prepared in BPE (biphosphate EDTA) buffer solution: 6.0 mM Na_2HPO_4, 2.0 mM NaH_2PO_4, 1.0 mM Na_2EDTA; pH 7.0. All buffer solutions were prepared from purified water (resistivity 18 MΩ cm) and biochemistry-grade chemicals. The buffer solutions were filtered through a PVDF membrane filter (pore size 0.45 µm) prior to use.

3. Synthesis

2,2'-[(1"-hydroxy-4"-methyl-(E)-2",6"-phenylene)]-bis-(quinolizinium) dibromide (**3a**)

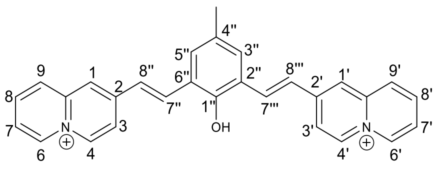

To a refluxing solution of 2-hydroxy-5-methylisophthalaldehyde (100 mg, 0.60 mmol) and 2-methylquinolizinium (550 mg, 2.45 mmol, 4.0 equiv.) in acetonitrile (10 ml) was added piperidine (57 mg, 66 µL, 0.7 mmol, 1.5 equiv.). The mixture was stirred under reflux for 24 h. After cooling the mixture to room temp., the reaction mixture was added dropwise to a stirred solution of diethyl ether (30 ml), and a green solid precipitated. The solid was filtered, washed with ether, and dried in a vacuum to afford a dark green solid that was further purified by column chromatography (SiO_2, $CHCl_3$/MeOH, 85:15, R_f = 0.32) to give the product **3a** as dark green amorphous solid (150 mg, 0.31 mmol, 70%); mp >300° C(dec.). – ^{1}H NMR (600 MHz, DMSO-d_6): δ = 2.36 (s, 3 H, CH_3), 7.53 (d, 2 H, ^3J = 16.2 Hz, 7"-H, 7'''-H, 8"-H, 8'''-H), 7.66 (s, 2 H, 3"-H, 5"-H), 7.97 (t, 2 H, ^3J = 6.9 Hz, 7-H, 7'-H), 8.19 (d, 2 H, ^3J = 16.2 Hz, 8"-H, 8'''-H, 7"-H, 7'''-H), 8.29 (t, 2 H, ^3J = 7.8 Hz, 8-H, 8'-H), 8.40 (d, 2

[1] a) A. Richards, T. S. Stevens. *J. Chem. Soc.* **1958**, 3067; b) O. F. Beumel, Jr.; W. N. Smith, B. Rybalka, *Synthesis* **1974**, *1*, 43; c) R. R. Gagne, C. L.Spiro, T. J. Smith, C. A. Hamann, W. R. Thies, A. D. Shiemke, *J. Am. Chem. Soc.* **1981**, *103*, 4073; d) B. Morgan, D. Dolphin, *J. Org. Chem*. **1987**, *52*, 5364.

H, ^3J = 6.6 Hz, 3-H, 3'-H), 8.46 (d, 2 H, ^3J = 8.4 Hz, 9-H, 9'-H), 8.56 (s, 2 H, 1-H, 1'-H), 9.26 (d, 2 H, ^3J = 6 Hz, 6-H, 6'-H), 9.30 (d, 2 H, ^3J = 6.6 Hz, 4-H, 4'-H). – ^{13}C NMR (150 MHz, DMSO-d_6): δ = 152.7 (C1'), 145.3 (C2, C2'), 142.8 (C9a, C9'a), 136.7 (C8, C8'), 136.5 (C4, C4', C6, C6'), 133.4 (C7", C7‴, C8", C8‴), 129.3 (C3", C5"), 129.2 (C4"), 126.8 (C9, C9'), 124.5 (C2", C6"), 123.9 (C8", C8‴, C7", C7‴), 122.6 (C1, C1', C7, C7'), 120.5 (C3, C3'), 20.2 (C-CH$_3$). – MS (ESI$^+$): *m/z* = 415 (100) [M – 2Br]$^{2+}$, 208 (36) [M–Br]$^+$. – El. Anal. for $C_{29}H_{24}N_2OBr_2 \times 2\ H_2O$ (612.4), calcd (%): C, 56.88; H, 4.61; N, 4.57, found (%): C, 56.88; H, 4.34; N, 5.09.

2,2'-[(1"-Methoxy-4"-methyl-(E)-2",6"-phenylene)]-bis-(quinolizinium) dibromide (**3b**)

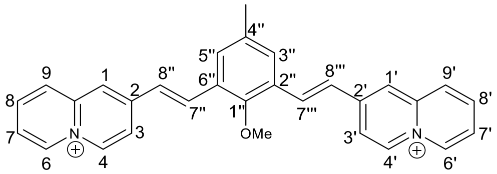

To a refluxing solution of 2-methoxy-5-methylisophthalaldehyde (50.0 mg, 0.28 mmol) and 2-methylquinolizinium (190 mg, 0.84 mmol, 3.00 equiv.) in acetonitrile (10 ml) was added piperidine (36 mg, 42 µL, 0.4 mmol, 1.5 equiv.). The reaction mixture was stirred at reflux for 20 h. After cooling to room temp. the reaction mixture was added dropwise to a stirred solution of diethyl ether (50 ml). The resulting green precipitate was further purified by column chromatography (SiO_2, $CHCl_3$/MeOH, 80:10, R_f = 0.33) to give the qualitative pure product **3b** as dark green amorphous solid (100 mg, 0.17 mmol, 60%); mp > 300° C (dec.). – ^{1}H-NMR (600 MHz, DMSO-d_6): δ 2.43 (s, 3H, -CH$_3$), 3.88 (s, 3H, -OMe), 7.65 (d, 2H, *J* = 16.8 Hz, 7"-H, 7‴-H), 7.81 (s, 2H, 3"-H, 5"-H), 7.99 (d, 2H, *J* = 16.8 Hz, 8"-H, 8‴-H), 8.01 (t, 2H, *J* = 6.6 Hz, 7-H, 7'-H), 8.33 (t, 2H, *J* = 7.8 Hz, 8-H, 8'-H), 8.51 (t, 4H, *J* = 7.8 Hz, 3-H, 3'-H, 9-H, 9'-H), 8.68 (s, 2H, 1-H, 1'-H), 9.32 (t, 4H, *J* = 6.3 Hz, 4-H, 4'-H, 6-H, 6'-H). – ^{13}C-NMR (150 MHz, DMSO-d_6): δ = 155.6 (C1"), 144.7 (C2, C2'), 142.8 (C9a, C9'a'), 136.9 (C8, C8'), 136.6 (C4, C4', C6, C6'), 134.2 (C4"), 131.8 (C7", C7‴, C8", C8‴), 129.4 (C3", C5"), 129.2 (C9, C9'), 126.9 (C2", C6"), 125.8 (C8", C8‴, C7", C7‴), 123.2 (C1, C1'), 122.9 (C7, C7'), 120.8 (C3, C3'), 63.6 (*C*–OCH$_3$), 20.6 (*C*–CH$_3$). – MS (ESI$^+$): *m/z* = 509 (100) [M – Br]$^+$. – El. Anal. for $C_{30}H_{26}N_2OBr_2 \times 3\ H_2O$ (644.4), calcd (%): C, 55.92; H, 5.01; N, 4.35, found (%): C, 55.64; H, 4.63; N, 4.53.

4. Absorption and emission spectra in media with different viscosity

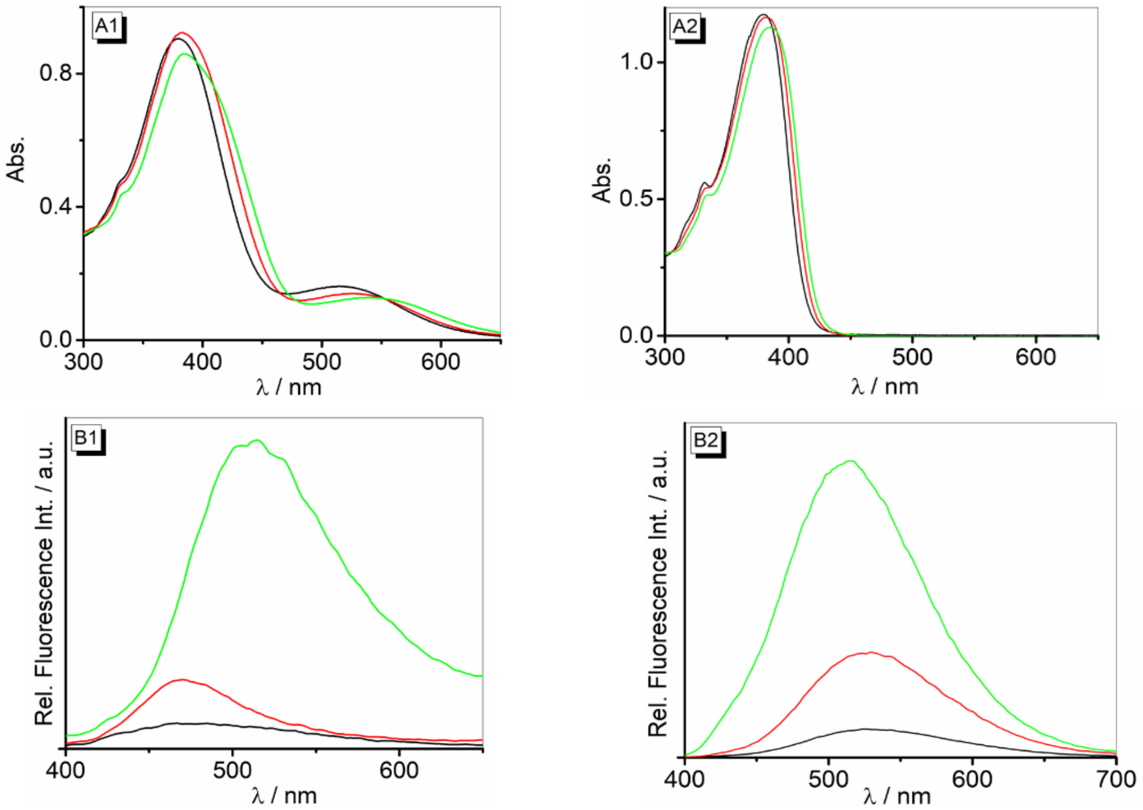

Figure S1. Absorption (A) and emission spectra (B) of **3a** (1) and **3b** (2) (c = 20 μM) in glycerol–H_2O mixtures; wt.% of glycerol in glycerol–H_2O mixtures: 0 (black), 50 (red), 100 (green).

5. Acid-base titrations

i) Acid-base titrations were performed in Britton–Robinson buffer solution, as obtained from phosphoric acid, boric acid, and sodium acetate (0.04 M each) in water, adjusted to a particular pH value by addition of aq. solution of NaOH (2 M) or aq. HCl (2 M).[2] Following each addition step, the pH and the absorption spectra were recorded. The absorption at a particular wavelength was plotted versus the pH of the solution, and the resulting isotherm was used to determine acidity constant pK_a by numerical fitting to the Henderson-Hasselbalch equation.[3]

The pKa value and the corresponding error of **3a** were obtained by numerical fit of the titration isotherms to the "dose resp" function of equation 1.

$$y = A_1 + (A_2 - A_1) / (1 + 10\text{^}((LOGx_0\text{-}x)*p)) \qquad \text{(Eq. 1)}$$

[2] H. T. S. Britton, R. A. Robinson, *Chem. Soc.* **1931**, 458.
[3] J. Polster, H. Lachmann, *Spectrometric Titrations: Analysis of Chemical Equilibria*, VCH, Weinheim, 1989.

A_1 = Absorption at initial pH

A_2 = Absorption at final pH

$LOGx_0$ = pKa

p = Hill slope

ii) Emission spectra were recorded in aq. $HClO_4$ (11.8 M) solutions and in aq. NaOH (pH 8.5) solutions to assign the bands of **3a** and the conjugate base **3a**^{cB} (Figure S2). The energy of the 0-0 transitions were estimated by means of average between the wavenumbers corresponding to the maxima of absorption and emission (Eq. 2).

$$\tilde{\nu}_{00} = (\tilde{\nu}_{abs}{}^{max} + \tilde{\nu}_{fl}{}^{max}) / 2 \qquad \text{(Eq. 2)}$$

The excited-state acidity constant pK_a^* was estimated according to the Förster cycle (Eq. 3).

$$\Delta pK = 0.00209\,[\tilde{\nu}\,(cB) - \tilde{\nu}\,(Acid)] / cm^{-1} \qquad \text{(Eq. 3)}$$

$\tilde{\nu}$ (cB) = Energy of the 0-0 transition of the conjugate base, in wavenumbers

$\tilde{\nu}$ (Acid) = Energy of the 0-0 transition of the acid, in wavenumbers

$\tilde{\nu}$ (**3a**) = 22887 cm^{-1}

$\tilde{\nu}$ (**3a**^{cB}) = 16418 cm^{-1}

pK_a^* = –6.4

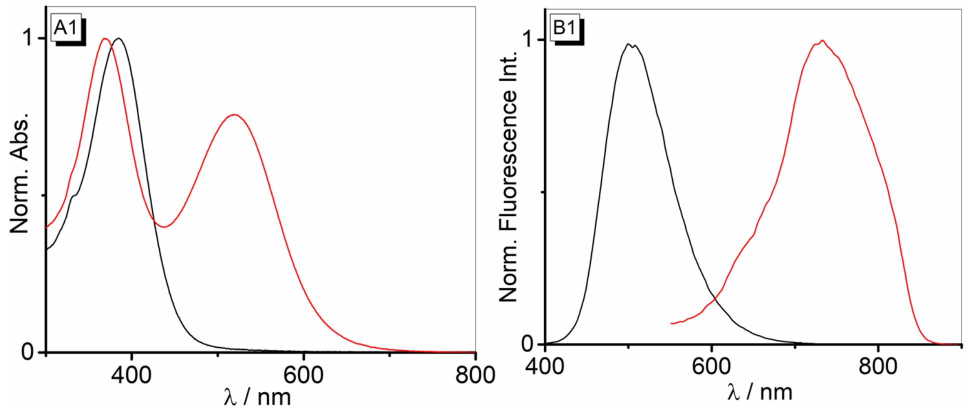

Figure S2. A1: Normalized absorption (A1) and emission spectra (B1) of **3a** (c = 20 µM, λ_{ex} = 376 nm) in aq. $HClO_4$ (11.8 M) (black) and in Britton-Robinson buffer at pH 8.5 (red, λ_{ex} = 520 nm).

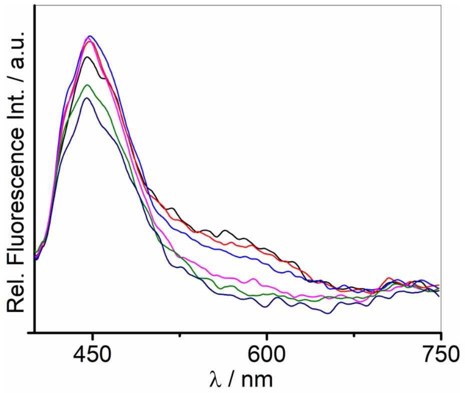

Figure S3. Fluorescence spectra of **3a** (c = 20 µM) at different pH values; from pH 2.0 (black line) to pH 10 (blue line); λ_{ex} = 376 nm.

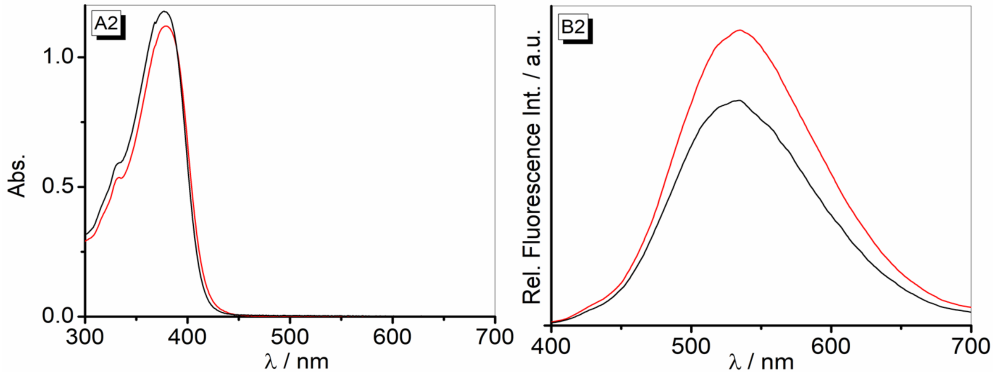

Figure S4. UV-vis (A) and Fluorescence spectra (B) of **3b** (2) (c = 20 µM) in highly acidic aq. $HClO_4$ (11.8 M) (red) and in Britton-Robinson buffer at pH 8.5 (black).

iii) The assignment of the emission bands of **3a** were further confirmed by experiments in 2,2,2-trifluoroethanol (TFE), i.e. a protic solvent with no proton-acceptor properties.[4]

Aliquots of a stock solution of **3a** in MeOH (c = 1.0 mM) were evaporated under a stream of nitrogen and redissolved in the solvent mixture of trifluoroethanol/ethanol with increasing EtOH content from 20% to 100%. Fluorescence spectra were recorded with excitation and emission slits of 5 nm and an excitation wavelength λ_{ex} = 440 nm.

[4] M. J. Kamlet, J. L. M. Abboud, M. H. Abraham, R. W. Taft, *J. Org. Chem*. **1983**, *48*, 2877.

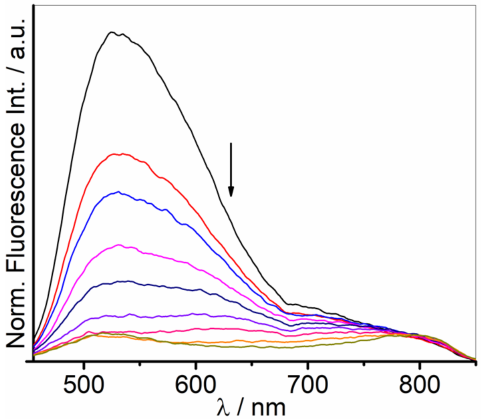

Figure S5. Fluorescence spectra of **3a** (c = 20 µM) in trifluoroethanol/ethanol mixtures with increasing fraction of EtOH: 20% (black), 25% (red), 30% (blue), 35% (magenta), 40% (navy), 50% (violet), 60% (pink), 80% (orange), 100% (yellow).

In TFE, **3a** exhibits a strong emission (Φ_{fl} = 0.35) with maximum at 530 nm. On addition of EtOH, which is a proton acceptor, this emission band decreased and an additional emission band developed at 790 nm corresponding to the emission of the conjugate base **3a**$^{\mathbf{cB}}$.

6. Determination of fluorescence quantum yield

Aliquots of a stock solution of derivatives **3a** and **3b** in MeOH (c = 1.00 mM) were evaporated under a stream of nitrogen and redissolved in the respective solvent for fluorimetric analysis. The relative fluorescence quantum yields, Φ_{fl}, of **3a** and **3b** were determined according to Eq. 4[5] under identical conditions (detection wavelength, excitation wavelength, detector voltage, slit bandwidths, collection rate).

$$\Phi_{fl,x} = \Phi_{fl,s} \, (F_x / F_s)(A_s / A_x)(n_x^2 / n_s^2) \qquad \text{(Eq. 4)}$$

The indices x and s indicate the analyte (x) and standard (s) solution.

F = area under the emission curve, A = absorbance at the excitation wave length,

n = index of refraction of the solvent.

Perylene in EtOH was used as standard (Φ_{fl} = 0.92) (error ~ 10%).[5]

7. Photometric and fluorimetric DNA titrations

For photometric and fluorimetric titration of **3a** and **3b** with ct DNA, aliquots of a stock solution in MeOH (c = 1.00 mM) were evaporated under a stream of nitrogen, redissolved in DMSO (5% v/v) and BPE buffer to obtain a ligand concentration of c = 20 µM. The DNA solutions contained also the ligand at the same concentration in order to avoid dilution effects. For the detection of emission spectra the excitation and emission slits were adjusted to 5 nm. The spectra were smoothed with the implemented moving average function by a factor of 5. Samples of the ligand solutions were

[5] a) B. Valeur, M. N. Berberan-Santos, *Molecular fluorescence. Principles and applications*, Wiley-VCH, Weinheim, 2nd ed., 2012; b) G. A. Crosby, J. N. Demas, *J. Phys. Chem*. **1971**, *75*, 991.

placed into quartz cells and absorption or emission spectra were recorded. The titrations were performed up to saturation. All spectrometric titrations were performed at least two times to ensure reproducibility. The binding constants were determined from plots of the absorption at a given wavelength versus relative DNA concentration, c_{DNA} / c_{ligand}, and fitting of the experimental binding isotherm (Figure S7) to the theoretical model (Eq. 4).[6]

$$\frac{I}{I_0} = 1 + \frac{Q-1}{2}\left(A + xn + 1 - \sqrt{(Q + xn + 1)^2 - 4xn}\right) \qquad \text{(Eq. 5)}$$

$Q = I / I_0$ = the minimal absorbance in the presence of excess ligand

n = number of independent binding sites per DNA

$A = 1 / (K_b \times c_{ligand})$

$x = c_{DNA} / c_{ligand}$ = titration variable

Standard deviations (SD) of K_b values were calculated from equation 6.

$$\text{SD}(K_b) = \{(\text{SD of A / A}) / \text{A}\} / c_{lig} \qquad \text{(Eq. 6)}$$

The difference of SD values for Kb values of **3a** and **3b** originates in in different contributions of the A value for **3b** that is bit of higher as compared to the A value for **3a**. Moreover, the A parameter is a kind of intersection.

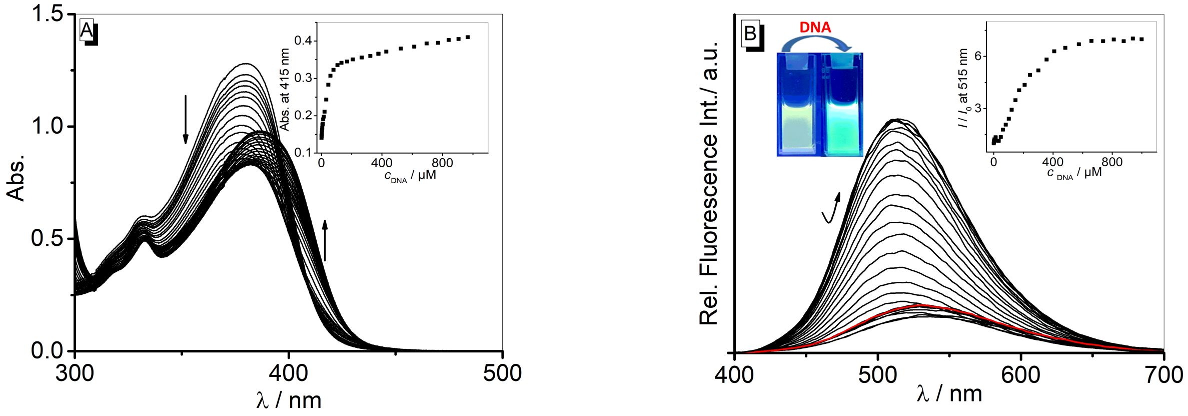

Figure S6. Photometric (A) and fluorimetric titration (B) of **3b** with ct DNA (c = 2.7 mM in base pairs) in BPE buffer (10 mM, pH 7.0; with 5% v/v DMSO, $c_{Lig.}$ = 20 µM); T = 20 °C; λ_{ex} = 380 nm. Arrows indicate the changes of bands on addition of DNA. Insets A: Plot of the absorption (A) or relative emission (B) vs. c_{DNA} and emission color of **3b** (B).

[6] F. H. Stootman, D. M. Fisher, A. Rodger and J. R. Aldrich-Wright, *Analyst* **2006**, *131*, 1145.

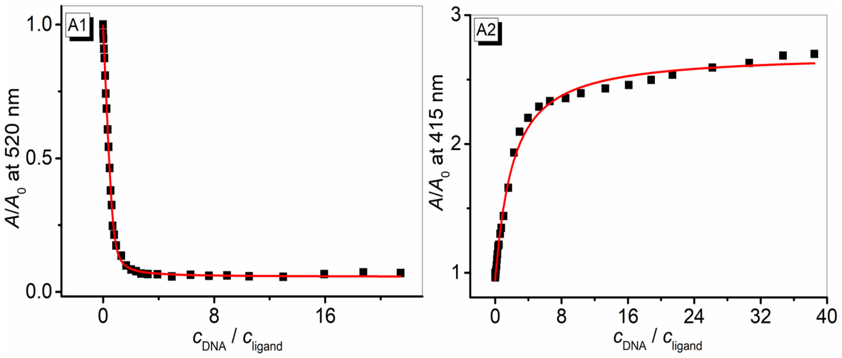

Figure S7. Fitting curves of binding isotherms, i.e. plot of A / A_0 vs. c_{DNA} / c_{ligand}, from spectrophotometric titrations of **3a** (A1) and **3b** (A2) for the determination of binding constants (K_b) with ct DNA. Red lines represent the best fits to the theoretical model.

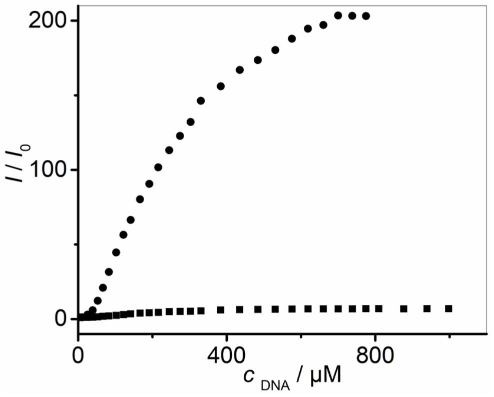

Figure S8. Plot of relative emission intensity I/I_0 of **3a** (circles) and **3b** (squares) (c = 20 µM) versus concentration of ct DNA.

8. CD- and LD-spectroscopic experiments

CD and LD spectra were recorded in BPE buffer solution at different ligand-DNA ratios (LDR) at fixed DNA concentration (c_{DNA} = 50.0 µM). The CD and LD measurements were performed at *LDR* = 0, 0.3, 0.5, 0.6, 0.8, 1.0 with band width of 1 nm, recording speed of 1 nm/s and time per data point of 0.5 seconds. *Flow*-LD spectra were recorded on CD spectrometer equipped a High Shear Couette Cell Accessory (Applied Photophysics). The LD samples were recorded in a rotating couette with a shear gradient of 1200 s^{-1}.

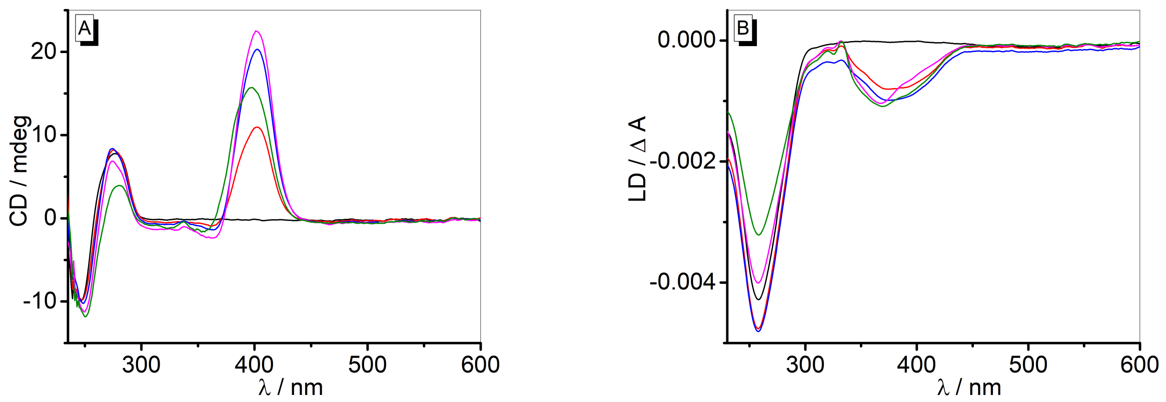

Figure S9. CD (A) and *flow*-LD spectra (B) of ct DNA (*c* = 50 µM) in the absence and presence of **3b** at *LDR* = 0 (black), 0.3 (red), 0.5 (blue), 0.8 (magenta), 1.0 (green) in BPE buffer solution (10 mM, pH 7.0; with 5% v/v DMSO).

9. NMR spectra

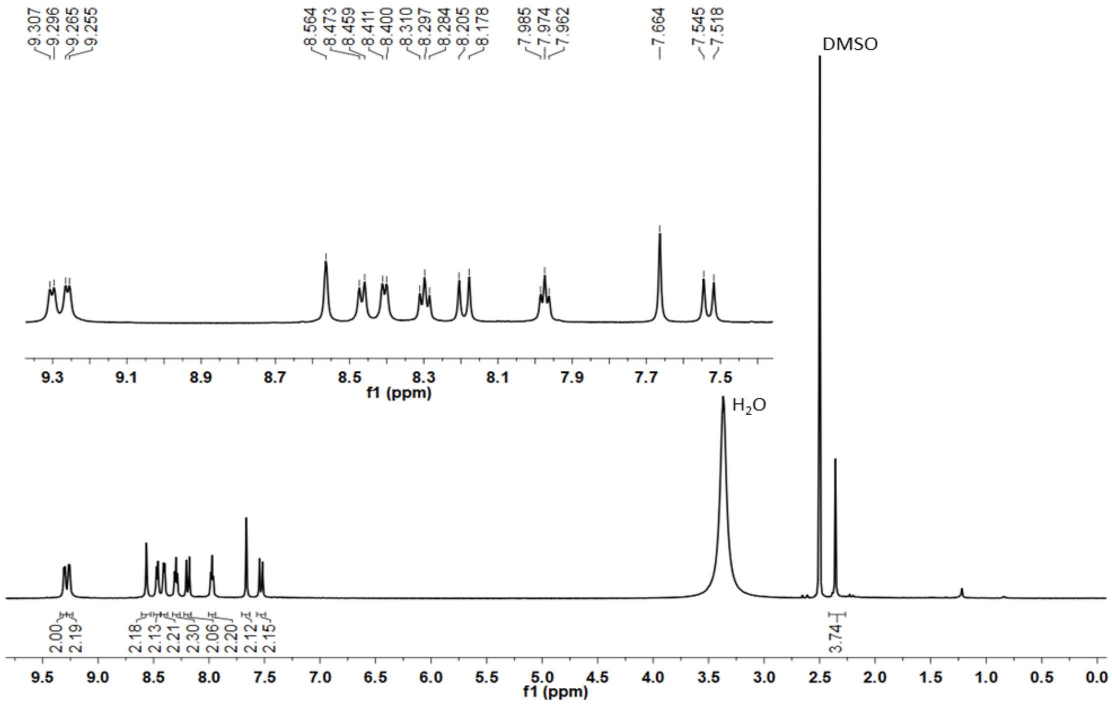

Figure S10. ^{1}H-NMR spectrum (600 MHz) of **3a** in DMSO-d_6 , Inset: Magnified range of aromatic protons.

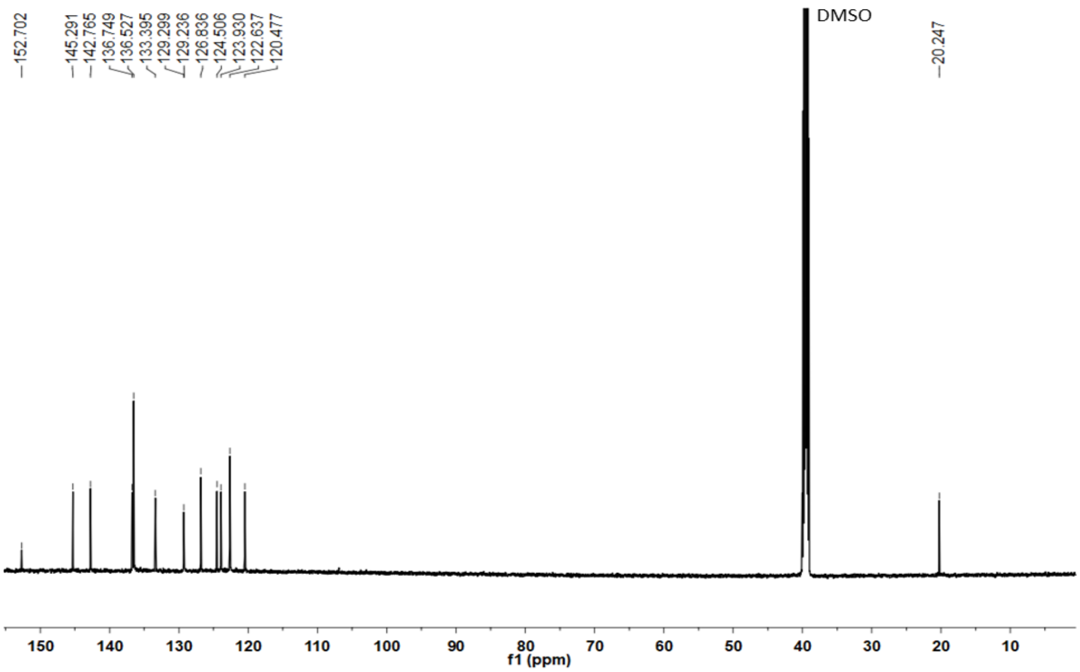

Figure S11. ^{13}C-NMR spectrum (150 MHz) of **3a** in DMSO-d_6.

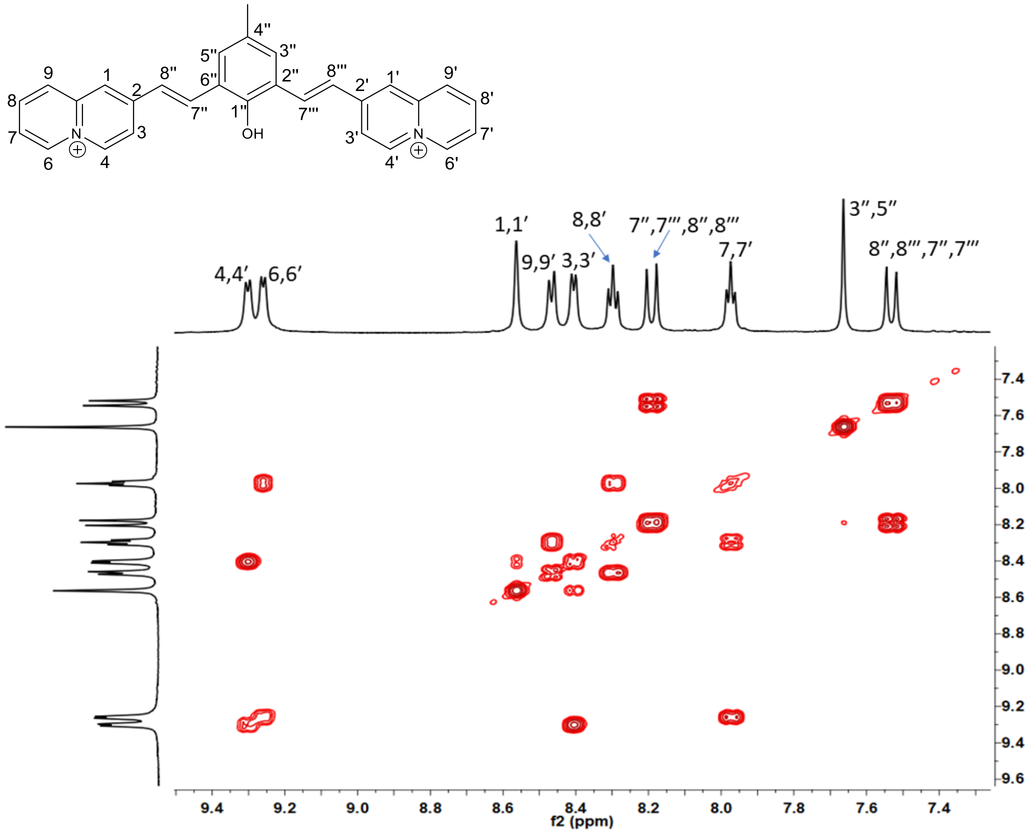

Figure S12. HH-COSY spectrum (600 MHz) of **3a in** DMSO-d_6

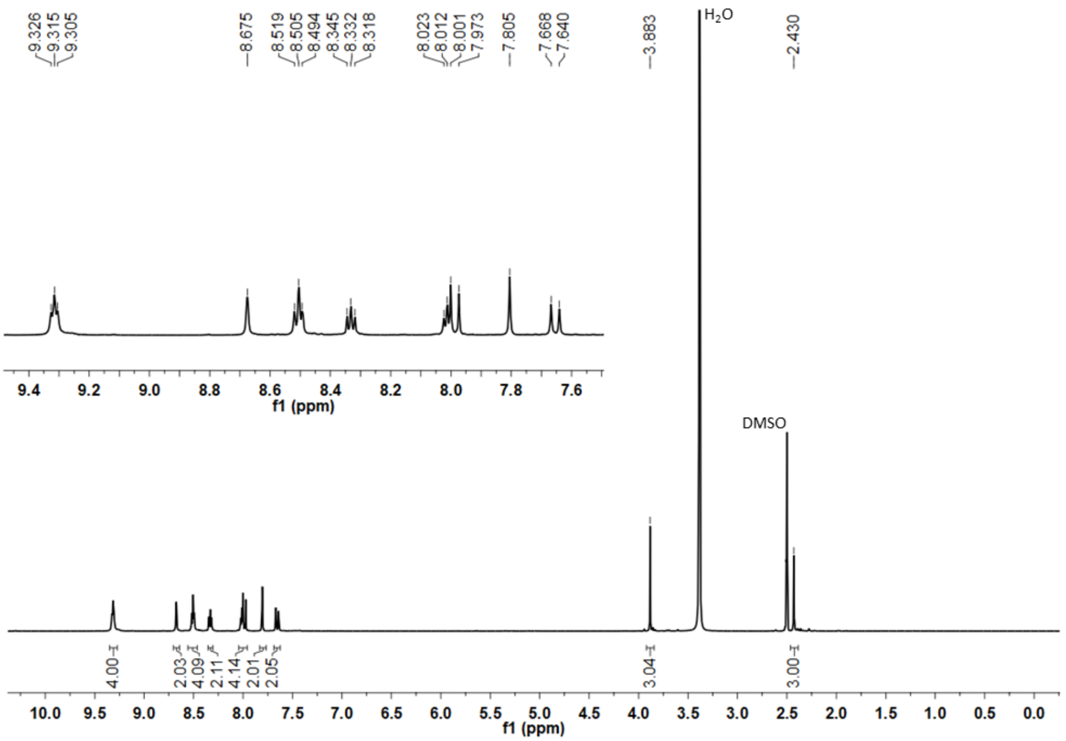

Figure S13. ^{1}H-NMR spectrum (600 MHz) of **3b** in DMSO-d_6 (Inset: Expansion with peak picking).

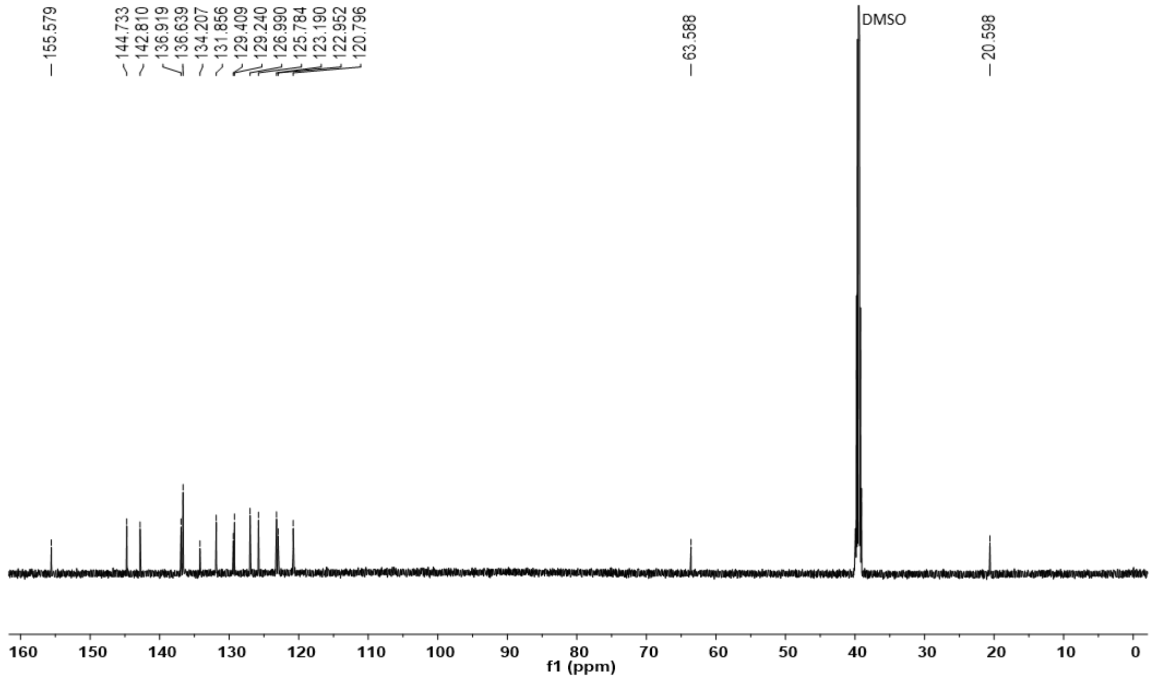

Figure S14. ^{13}C-NMR spectrum (150 MHz) of **3b** in DMSO-d_6.

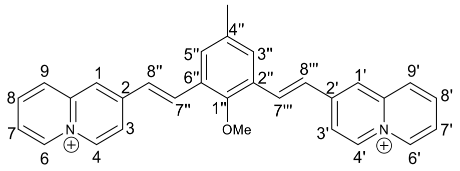

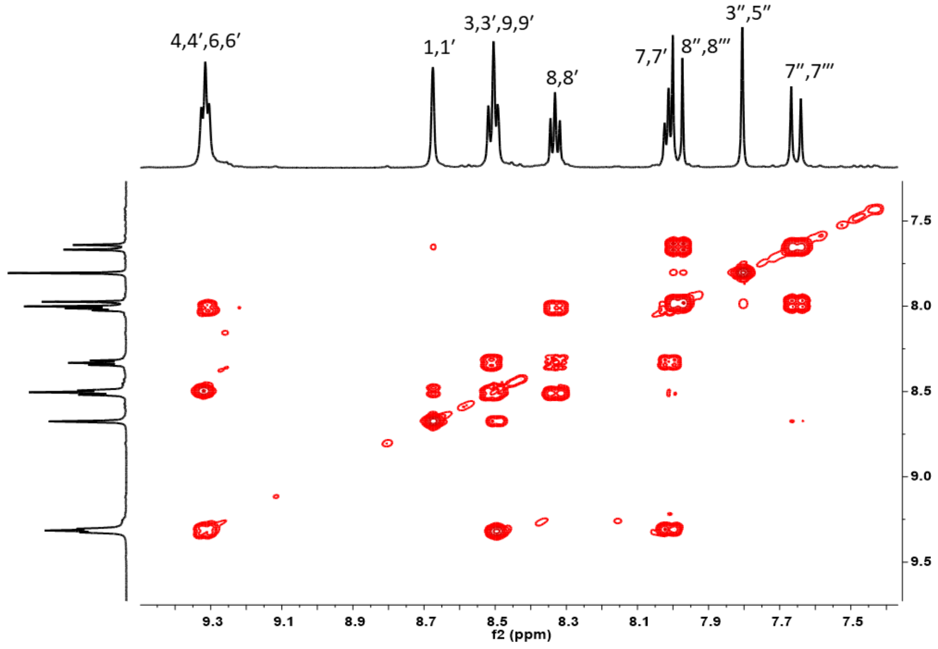

Figure S15. HH-COSY spectrum of **3b** in DMSO-d_6

10. NIH 3T3 cell culture and fluorescence staining

The NIH 3T3 mouse fibroblasts[7] were cultured at standard conditions (37°C, 5% CO_2) in Dulbecco's modified Eagle medium (DMEM high glucose; Gibco, Thermo Fisher Scientific) supplemented with 10% fetal bovine serum (Gibco, Thermo Fisher Scientific), 2 mM L-glutamine (Gibco, Thermo Fisher Scientific), 100 U mL^{-1} penicillin (Gibco, Thermo Fisher Scientific) and 100 µg mL^{-1} streptomycin (Gibco, Thermo Fisher Scientific). Subcultivation of cells was performed by treatment with 0.25% Trypsin/EDTA of cell culture layers washed with PBS (Lonza, Belgium). Released cells were collected and centrifuged (270 x g for 4 min) in a conical tube, counted with a Neubauer improved cell counting chamber (Brand, Wertheim, Germany) and seeded at a density of 10.000 cells cm^{-2} on glass cover slips (Menzel Gläser 20 x 20 mm, thickness 0.17 mm) in 6-well plates (Standard F, Sarstedt AG. & Co.). Cells were cultured to a 60% confluency before fluorescent labeling. If cells were fixed before the fluorescent labeling with **3a**, they were washed gently three times with pre-warmed (37 °C) PBS (Lonza, Belgium), fixed with 4% paraformaldehyde in PBS for 20 min at 21 °C and washed again three times with PBS at 21 °C.

If the fluorescent labeling with **3a** was performed with living cells, the culture medium was removed only once.

For the fluorescent labeling, cells were incubated with 2.5 µM **3a** as a final concentration (**3a** stock concentration: 1.0 mM in DMSO) in either pre-warmed cell culture media for staining of living cells or PBS for staining of fixed cells for 1 h at 37 °C and 5% CO_2 (living cells) or at room temperature (fixed cells). As controls, cells have been in parallel incubated with cell culture media or PBS only or with the same final concentration of DMSO [0.25 % (v/v)].

After staining of living cells they were washed two times with pre-warmed (37°C) PBS and the fixation protocol was performed as described above.

For counter staining with Hoechst 33258 (Carl Roth) as a gold standard, cells were optionally incubated with 1 µg/mL Hoechst 33258 in PBS for 15 min at room temperature. Before final sample embedding with Mowiol® 4-88 (Carl Roth) samples were washed three times with PBS and once with Milli-Q water (from a Millipore Direct Q8 system, advantage A10 system, Schwalbach, with Millimark Express 40 filter, Merck, Germany).

Epifluorescence microscopy for cell analysis was done with an Axiovert 135 microscope equipped with an AxioCamMRm and Zen 2.3lite software and the following filter sets (49, 09, Lumar 15); 1) λ_{ex} = 320–390 nm, λ_{em} = 420–470 nm; 2) λ_{ex} = 450–490 nm, λ_{em} >515 nm; 3) λ_{ex} = 540–552 nm, λ_{em} > 590 nm; Carl Zeiss MicroImaging GmbH, Jena, Germany).

The camera exposition time for filterset 1) was 4000 ms, for 2) 10000 ms and for 3) 20000 ms. Brightness and contrast were optimized by the "auto" adjustment of the Zen2.3 lite software without information loss.

[7] J. L. Jainchill, S. A. Aaronson, G. L. Todaro, *J. Virol.* **1969**, *4*, 549.

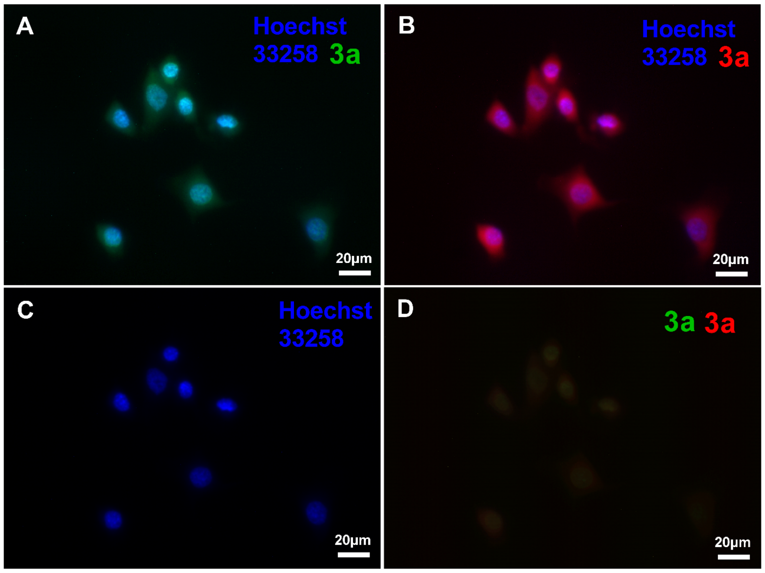

Figure S16. (Complementary Figure to Figure 4 showing the same image section): Epifluorescence microscopy images of NIH 3T3 mouse fibroblasts after fixation, stained with **3a** (2.5 µM in PBS) for 1 h and counterstained with the standard nuclear staining dye Hoechst 33258. The images show pseudo coloring of the fluorescence emission resulting from three different filter sets: A) Overlay of green channel: λ_{ex} = 450–490 nm, λ_{em} >515 nm (**3a**) with blue channel: λ_{ex} = 320–390 nm, λ_{em} = 420–470 nm (Hoechst 33258) B) Overlay of red channel: λ_{ex} = 540–552 nm, λ_{em} > 590 nm and blue channel: λ_{ex} = 320–390 nm, λ_{em} = 420–470 nm (Hoechst 33258) C) Hoechst 33258 stain alone in the blue channel and D) Overlay of red and green channels of the raw pictures without any automatic brightness / contrast adjustment via the ZEN-Software (for comparison with Figure 4C). Labeling **3a** stands for both the ligand **3a** and its conjugate base **3a**^{cB} compound. Scale bar: 20 µm.

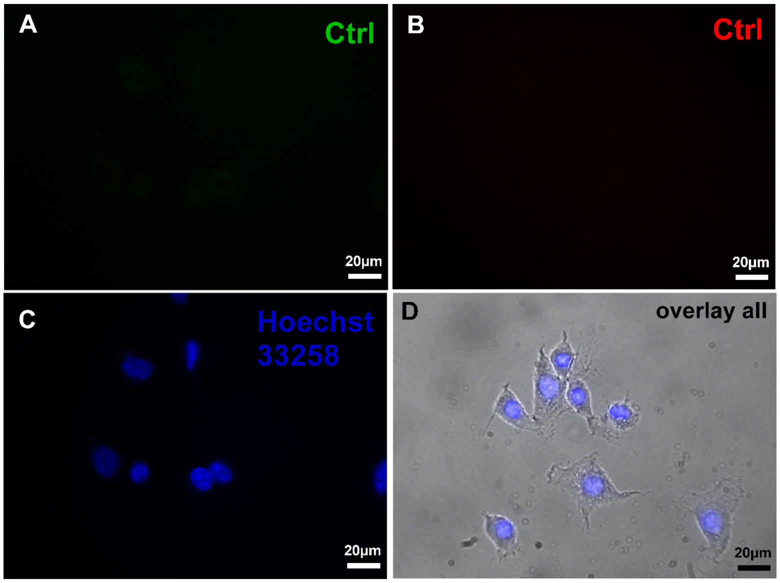

Figure S17. Epifluorescence microscopy images of NIH 3T3 mouse fibroblasts after fixation. The cell nuclei were stained with a standard Hoechst 33258 stain solution. The background fluorescence in the red and green channel are negligibly low. Corresponding exposition times as well as automatic brightness and contrast adjustment were applied. The images show pseudo coloring of the fluorescence emission resulting from three different filter sets: green channel: λ_{ex} = 450–490 nm, λ_{em} >515 nm; B) red channel: λ_{ex} = 540–552 nm, λ_{em} > 590 nm; C) Blue channel: λ_{ex} = 320–390 nm, λ_{em} = 420–470 nm (Hoechst 33258) ; D) Overlay of blue channel with the corresponding bright field image.

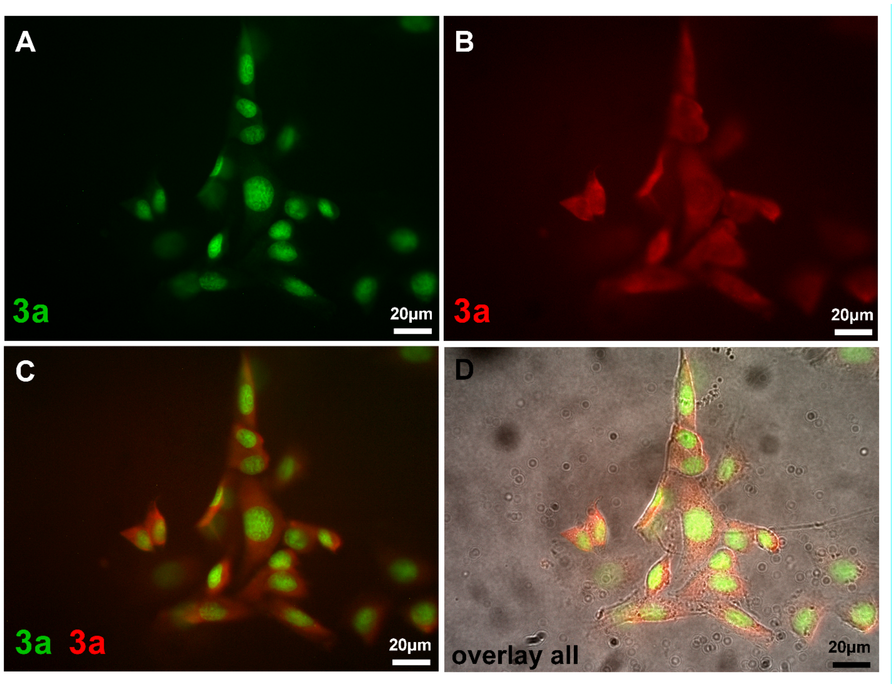

Figure S18. Epifluorescence microscopy images of NIH 3T3 mouse fibroblasts, which were stained with **3a** (2.5 µM in PBS) for 1 h in cell medium prior to fixation with 4 % PFA. The images show pseudo coloring of the fluorescence emission resulting from three different filter sets: A) green channel: λ_{ex} = 450–490 nm, λ_{em} >515 nm; B) red channel: λ_{ex} = 540–552 nm, λ_{em} > 590 nm; C) Overlay of red and green channel; D) Overlay of red and green channel with the corresponding bright field image. Labeling 3a stands for both the ligand 3a and its conjugate base $3a^{cB}$ compound.

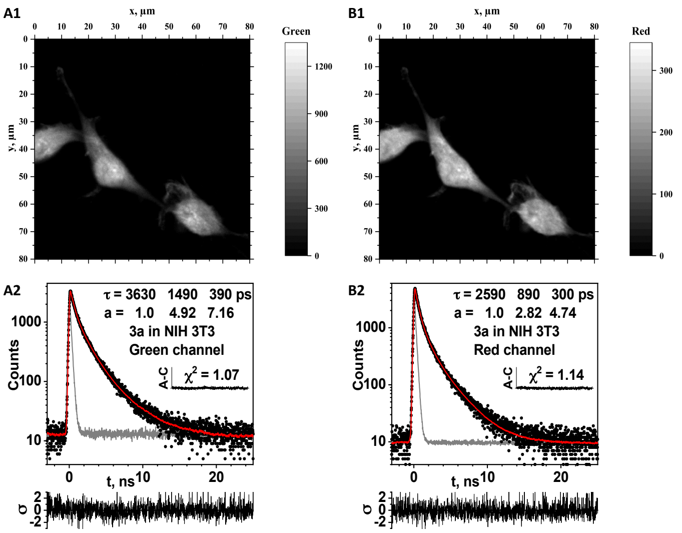

Figure S19. Confocal fluorescence microscopy images of NIH 3T3 mouse fibroblasts after fixation and incubation with **3a** (2.5 µM) for 1 h. Excitation wavelength λ_{ex} = 485 nm, emission wavelength λ_{em} = 515–652 nm (A1) and 652–732 nm (B1). Semi-logarithmic plot of the TCSPC triple-exponential fluorescence decays in corresponding images A1 and B1 at λ_{em} > 515 nm (A2) and 652–732 nm (B2) (black dots) together with the corresponding instrument response function (gray line) and calculated fits (red line) at the time resolution of 16 ps. In the A2 and B2 panels, the weighted deviations (σ) and the autocorrelation function (A-C) plots as well as the decay times (τ), their amplitudes (a) and χ^2 fit quality parameter are shown.

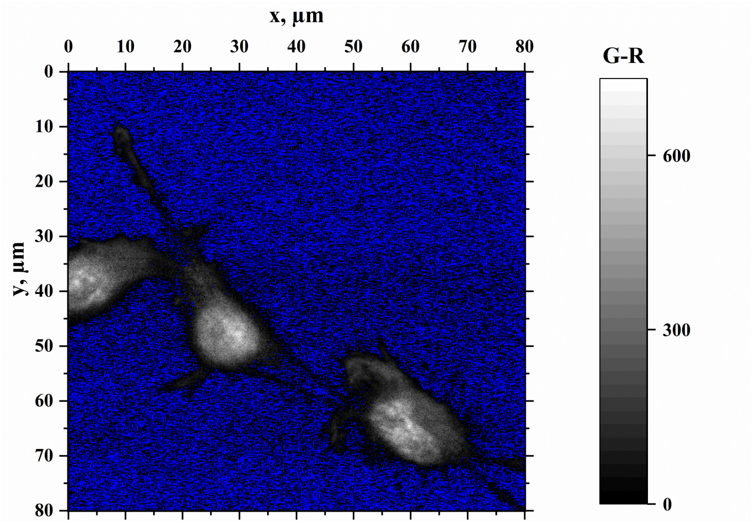

Figure S20. Confocal fluorescence intensity difference image resulting from **G** – 2**R** for NIH 3T3 mouse fibroblasts after fixation and incubation with **3a** (2.5 µM) for 1 h. Here **G** (**R**) is the image from Fig. 5, A1 (B1). Excitation wavelength λ_{ex} = 485 nm, emission wavelength λ_{em} = 515-652 nm (**G**) and 652–732 nm (**R**). A close to zero negative intensity of background is shown in blue.
